# A moderated multilevel multiple mediation model of the effect of class teacher communication style on adolescents' school adjustment: the roles of basic psychological needs and psychological *suzhi*

**DOI:** 10.3389/fpsyg.2025.1678864

**Published:** 2025-12-18

**Authors:** Songyan Dong, Jihui Wu

**Affiliations:** School of Education, China West Normal University, Sichuan, China

**Keywords:** adolescence, basic psychological needs, psychological *suzhi*, school adjustment, teacher communication style

## Abstract

**Aim:**

This study investigated the relationship between class teacher communication style and adolescents' school adjustment, with a particular focus on the mediating role of basic psychological needs and the serial mediating and moderating effects of psychological *suzhi*.

**Methods:**

A total of 2,578 adolescents from 51 classes across four schools participated in a questionnaire survey, yielding 2,418 valid responses. The sample included students from both rural and urban schools, ranging from grades 7 to 11. A moderated multilevel multiple mediation model was employed, in which basic psychological needs (autonomy, relatedness, and competence) and psychological *suzhi* formed a serial mediation pathway linking teacher communication style to school adjustment. Mediation and moderation effects were tested using the bias-corrected bootstrap method in Mplus.

**Results:**

Results showed that (1) both dimensions of teacher communication style significantly and positively predicted adolescents' school adjustment at both the student and class levels; (2) each of the three basic psychological needs independently mediated this relationship, with hierarchical differences in the mediation effects; (3) psychological *suzhi* also served as an independent mediator at the student level; (4) the serial mediation pathway through basic psychological needs and psychological *suzhi* was largely supported; and (5) psychological *suzhi* stably moderated the second stage of the competence-related mediation pathway at the student level.

**Conclusion:**

A communication style characterized by both communion and agency was more conducive to adolescents' school adjustment. Both basic psychological needs and psychological *suzhi* mediated the relationship between teacher communication style and school adjustment, with a serial mediation pathway also observed, albeit with differences across need types and analytical levels. Higher levels of psychological *suzhi* reduced adolescents' dependence on the satisfaction of competence needs, indicating a potential resource substitution effect.

## 1 Introduction

The class teacher communication style are considered key factors influencing adolescents' psychological development and adjustment (Jiang, 2002). Basic psychological needs (BPNs) and psychological *suzhi* are significantly associated with adolescents' school adjustment (Zhao et al., 2021b; Ratelle and Duchesne, 2014). However, empirical research on the relationships between class teacher communication style and BPNs or psychological *suzhi* remains limited. Additionally, the aggregation of different dimensions of BPNs may obscure the distinct effects of these needs. Therefore, this study aims to explore the impact of class teacher communication style on Chinese adolescents' school adjustment and to examine the roles of different dimensions of BPNs and psychological *suzhi* in this relationship.

### 1.1 Teacher communication style of class teachers and school adjustment

Ecological systems theory suggests that individual development takes place within a framework of interrelated environmental systems, which interact to influence psychological, behavioral, and developmental processes (Bronfenbrenner, 1992). Bronfenbrenner classified these systems into five levels, ranging from the most immediate to the broader influences: microsystem, mesosystem, exosystem, macrosystem, chronosystem.

As one of the primary living environments for adolescents, schools represent a critical context influencing their development. Within the framework of China's primary and secondary education system, class teachers are pivotal figures in the school environment. They are the teachers who interact most closely with students and serve as leaders of the teaching team, daily managers of the class, and facilitators of home-school communication, as well as student mentors. The official stance of China's Ministry of Education defines the role of class teachers as striving to become “life mentors for students.”

Class teachers have a significant impact on adolescents' psychological adjustment and development. Furthermore, numerous studies in China have examined the teacher-student relationship between class teachers and adolescents, highlighting its critical role in adolescents' psychological development and adjustment (Ai et al., 2024).

Furthermore, ecological systems theory posits that the influence of external systems on individuals often occurs through interaction, with direct interaction serving as the core pathway affecting psychological development (Bronfenbrenner, 1992). Teacher-student interaction encompasses all forms of mutual actions and influences between teachers and students and is the primary means through which teachers exert influence on students (Pianta, 2016). Research has shown that teacher-student interaction significantly impacts adolescents' school adjustment (Wang and Fletcher, 2017). Two primary mechanisms underpin this influence: first, the direct transmission of information during interactions; second, the formation of students' expectations based on teachers' long-term stable interpersonal patterns, which convey predictable psychological cues as a sustained, trait-driven environmental input (Lemay and Venaglia, 2016). This input meaningfully impacts adolescents' long-term school adjustment. (Mitchell et al. 2018) showed that student trust in teachers significantly and positively predicts their school identification. Given their multi-role responsibilities, class teachers' interaction pattern characteristics are likely to exert a more comprehensive and profound influence on adolescents compared to subject teachers.

However, existing research on teacher-student interaction has predominantly focused on subject teachers' interactions with students in teaching contexts or classrooms, which pertains to teachers' instructional behaviors (Patzak and Zhang, 2025; Ruiz-Alfonso et al., 2023). Meanwhile, existing research on class teachers has mainly explored their impact on adolescents' psychological adjustment from the perspectives of teacher-student relationship quality and behavioral strategies (Zhang et al., 2021; Ye et al., 2021), with little attention to the influence of stable interaction patterns class teachers exhibit during interactions on adolescents' adjustment.

Scholars have articulated teacher communication style from distinct perspectives (Ruby, 2025). However, given that the present study focuses on class teachers' multi-role responsibilities and the cross-contextual nature of their communication with students, and considering the adaptability of measurement items, the conceptualizations of this construct by (Wubbels and Levy 1993), as well as (Jiang 2002), are more aligned with the present research compared to those proposed by other scholars. (Wubbels and Levy 1993) distinguished teacher behaviors into two categories: instructional behaviors and interpersonal behaviors, defining teacher-student interaction within the scope of interpersonal behaviors, and emphasizing the concept of communication style. (Levy et al. 1992) framed it as “the characteristic of an individual's interpersonal behavior, belonging to the category of attitude.” Further specifying this in educational contexts, (Jiang 2002) defined it as “the attitude characteristics teachers exhibit in interpersonal interactions with students—whether in classroom teaching or extracurricular communication.” (Wubbels and Levy 1993) emphasized it should not be confused with generalized personality traits, which remain stable across all life contexts. (Jiang 2002), however, noted it is “conceptually close to personality”: it manifests as the externalization of teachers' personality in teacher-student interactions. Additionally, research by (Wubbels and Levy 1993) has shown that the mean changes in each dimension of student-perceived teacher communication style over a 4-month period were less than 0.01. Integrating Levy's focus on attitude, Jiang's emphasis on interaction-specificity and stability, and Wubbels' distinction from generalized personality, we define teacher communication style as a stable behavioral tendency of teachers in teacher-student interactions, which reflects teachers' personality traits within the context of teacher-student interactions but is not reducible to these generalized traits themselves.

Wubbels proposed and developed the teacher interpersonal circle (Wubbels and Levy, 1993; Wubbels et al., 2014), and the Questionnaire on Teacher Interaction (QTI), developed based on this model, is the most congruent tool for measuring teacher communication style (Wubbels and Levy, 1993; Burns and Van Bergen, 2025). The teacher interpersonal circle model, based on Leary's (1957) interpersonal circle, includes two fundamental dimensions: communion and agency. These dimensions were initially labeled as proximity and influence, later revised to affiliation and control, and ultimately refined to communion and agency. Communion emphasizes care, support, and prosocial behaviors exhibited by individuals, reflecting their ability and willingness to build connections with others. Agency focuses on autonomy and goal orientation, reflecting individuals' confidence, competence, dominance, and control over external circumstances (Abele and Wojciszke, 2007). Studies employing the QTI have demonstrated that teacher communication style are associated with students' outcomes (Mainhard, 2015; Den Brok et al., 2005).

School adjustment is a multidimensional and comprehensive concept, typically encompassing various aspects such as academics, school attitudes, emotions, interpersonal relationships, and behaviors. It is considered an important criterion for evaluating adolescents' mental health (Zhang and Jiang, 2006). Distinct from adaptability, which belongs to the domain of abilities, adjustment reflects individuals' psychological and behavioral responses to changes in their environment and physical/mental state, falling within the domain of states (Zhang and Jiang, 2006). This study defines school adjustment as the outcomes or manifestations of adolescents' interactions with the school environment and activities.

Numerous factors influence school adjustment, with teacher-student communication being a critical one. For instance, (Ruiz-Alfonso et al. 2023) demonstrated that teacher communication style significantly predicts students' passion and dedication, while (Cardenal et al. 2023) found that teaching style is significantly associated with teacher-student relationship quality. However, empirical research investigating the effect of class teacher communication style—not limited to instructional contexts—on adolescents' development and adjustment, particularly with mainland Chinese adolescents, remains limited. (Jiang 2002) explored the impacts of class teacher communication style on the school adjustment of students in elementary and secondary education and examined the mediating effect of classroom climate. Nonetheless, classroom climate, as an environmental variable, leaves questions about the role of internal psychological constructs such as psychological *suzhi* or BPNs in mediating this relationship, warranting further theoretical and empirical investigation. Additionally, given the dramatic societal changes in China over the past two decades, including significant improvements in teacher competencies and notable intergenerational differences in individual psychological traits, the dynamics of their relationship also require empirical validation. Therefore, this study proposed hypothesis:

*H1*: Communion and agency (the two dimensions of class teacher communication style) positively predict adolescents' school adjustment.

### 1.2 Mediating role of basic psychological needs

Basic psychological needs are a core concept in Self-Determination Theory (SDT) and are referred to as psychological nutrients that are crucial for individual adjustment, integration, and growth (Ryan, 1995). The theory also emphasizes the cross-cultural universality of these needs (Vansteenkiste et al., 2020). The three widely recognized BPNs are: autonomy, relatedness, and competence. According to the basic psychological needs model, BPNs mediate the relationship between the external environment and individual adjustment. A need-supportive environment helps satisfy BPNs, promoting psychological development and adjustment, whereas a need-thwarting environment can lead to frustration of BPNs, thereby hindering adjustment (Vansteenkiste et al., 2020).

Given the central role of environmental factors in shaping BPNs satisfaction, it is essential to identify specific interpersonal dynamics within educational settings that influence these needs. In this study, we focus on teacher communication style—a pervasive, stable dimension of the teacher-student environment—as the key environmental dynamic driving sustained BPNs satisfaction. To clarify the theoretical grounding of this focus, it is critical to distinguish between teacher communication style and teacher need-supportive behaviors (a core construct in SDT), as well as to elucidate the theoretical link between teacher communication style and BPNs.

Despite functional overlaps, the two constructs differ substantially in conceptual breadth. Teacher need-supportive behaviors are context-specific strategies targeting need satisfaction (Aelterman et al., 2019; Patall et al., 2018), often studied as discrete, instruction-focused actions (Wang and Zhao, 2022), which serve as core manifestations of one type of teachers' motivating style within the instructional domain (Aelterman et al., 2019). In contrast, teacher communication style—consistent with our prior definition as a stable behavioral tendency reflecting personality traits—encompasses broader, cross-contextual interaction patterns extending beyond formal instruction.

This distinction makes it more distinctly suited to our study. First, it aligns with class teachers' role as life mentors in Chinese education, whose responsibilities inherently entail cross-contextual interactions with students. Second, its stable nature provides sustained environmental input for psychological *suzhi* development (Zhang et al., 2017), directly resonating with our mediational focus on this construct.

Existing research underscores the necessity of this focus. Within SDT, existing studies related to teacher styles have primarily focused on the impact of teachers' motivating styles (such as autonomy support) on students' motivation, academic engagement, and academic achievement in instructional contexts, and have centered on the two types of BPNs—autonomy and competence (Wang and Zhao, 2022; Pulido et al., 2020; Behzadnia et al., 2018; Collie et al., 2019; Aelterman et al., 2019; Okada, 2021; Patzak and Zhang, 2025). Less attention has been paid to teachers' interpersonal behavioral styles.

Turning to the theoretical link between teacher communication style and BPNs, we draw on SDT. SDT posits that BPNs satisfaction relies on a stable, enduring interpersonal environment (Ryan and Deci, 2017). This aligns with teacher communication style's trait-like stability, enabling it to shape BPN satisfaction through two core dimensions:

First, teachers with high communion scores—characterized by greater helpfulness, friendliness, and understanding—consistently convey warmth and empathy, fostering students' predictable trust in reliable support. This directly fulfills relatedness needs (Ryan and Deci, 2017; Bridges, 2003) while indirectly enhancing autonomy by creating a safe space for perspective-sharing. (Cai et al. 2023) demonstrated that teacher empathy significantly and positively predicts both students' sense of school belonging and their reading achievement. (Mitchell et al. 2018) demonstrated a moderate positive correlation between student' trust in teachers and their perceptions of psychological safety.

Second, teachers with high agency scores—scoring high on the leadership and strictness subscales and low on the student freedom and uncertainty subscales—are more likely to provide students with explicit guidance and structured planning. Consistent with Lev Vygotsky's scaffolding theory (van de Pol et al., 2010), such guidance reduces ambiguity regarding what to do and how to do it in academic tasks. Empirically, both guidance and structure have been linked to higher academic achievement (Mouratidis et al., 2013; Horan and Carr, 2018).

By setting high standards and encouraging students to persist in overcoming challenges, these teachers foster an environment where students are more prone to experiencing enhanced competence need satisfaction (Ryan and Deci, 2017). This theoretical and contextual rationale further justifies our focus on teacher communication style within the SDT framework. It offers a more robust lens for explaining sustained BPNs satisfaction among adolescents.

In addition, in most studies related to BPNs, the three psychological needs exhibit moderate correlations with each other (Moltafet et al., 2018). Consequently, numerous studies have treated BPNs as an integrated latent variable (Zeng et al., 2022). However, (Vansteenkiste et al. 2020) emphasized the distinctiveness of each of the three BPNs and their incremental effects. Therefore, aggregating them into a single variable may confound and merge the effects of different BPNs, while treating them as a latent variable representing BPNs excludes the uniqueness of each need and results in a higher-order latent variable of BPNs. This approach is not conducive to exploring the mechanisms by teacher communication style influence adolescents' school adjustment, nor does it allow for examining the differential mediating roles of the three BPN dimensions in this relationship. Nevertheless, separately modeling each of the three needs may give rise to potential “apparent effects.” Therefore, this study modeled the three types of psychological needs separately as indicators of BPNs, while also constructing a combined model including the three needs for reference purposes, and proposed the following hypothesis:

*H2*: Basic psychological needs (autonomy, competence, and relatedness) mediates the associations between class teacher communication style (communion and agency) and adolescents' school adjustment.

### 1.3 Mediating role of psychological *Suzhi*

As a psychological construct originating in China, psychological *suzhi* refers to the process by which external stimuli are internalized into stable, fundamental, and implicit psychological qualities based on physiological conditions. These qualities exhibit foundational, derivative, evolutionary, and self-regulating functions and are strongly tied to individuals' adaptability, development, and creativity (Zhang et al., 2011). Psychological *suzhi* is theoretically defined as a three-dimensional integrated structure that covers cognitive, personality, and adaptive domains, forming a holistic psychological basis for adolescent development (Zhang et al., 2017).

Cognitive traits, as the fundamental components of psychological quality, refer to humans' reflective activities toward objective things (with direct involvement in cognitive operations), including general cognitive abilities (perceptual, analogical, comparative reasoning, serial relational thinking, abstract reasoning) and metacognitive abilities (awareness, planning, monitoring) that support individuals' information processing and problem-solving; personality traits, the core content of psychological quality (with motivational/regulatory functions), are individual psychological manifestations in engaging with objective things, covering aspirational level, independence, perseverance, intellectual curiosity, self-control, etc., and driving behavior while regulating emotions; adaptive capacity, a key factor reflecting derivative functions in psychological quality, is individuals' ability to alter themselves or the environment for harmony during socialization, integrating cognitive and personality traits in adaptation-development-creation and including physical-mental coordination, emotional/learning/interpersonal adaptability, and frustration tolerance to enable response to environmental changes (Zhang et al., 2011; Hu et al., 2017; Zhang et al., 2000).

From the perspective of the formation and development mechanism of psychological *suzhi*, psychological *suzhi*'s formation relies on the internalization of sustained external stimuli into stable psychological qualities (Zhang et al., 2011). Based on the cultural-historical activity theory, (Zhang et al. 2013) proposed that internalization is the basic mechanism for the formation of psychological *suzhi*, and practical activities such as interpersonal interaction are the main sources for the formation and development of psychological *suzhi*. (Wang and Zhang 2012a) proposed the relationship model between psychological *suzhi* and mental health, which comprehensively elaborates on the connotation, development, and mechanisms of psychological *suzhi*. According to this model, environmental factors influence individuals' psychological *suzhi* through active selection and passive acceptance. As a stable interpersonal behavioral tendency, class teacher communication style perfectly meets this sustained stimulus requirement—its dual dimensions affect the internalization and development of psychological *suzhi* through daily and repeated interactive practices.

More specifically, the friendliness and understanding exhibited by teachers with high communion scores create a psychologically safe atmosphere for adolescents, with positive impacts across three key dimensions of psychological *suzhi*: First, in terms of cognitive traits, teachers' encouraging feedback stimulates students' metacognitive awareness. (Kyriakides et al. 2020) demonstrated that teachers' questioning strategies are associated with students' metacognitive prediction and evaluation capacities. Second, regarding personality traits, sustained care and empathy from such teachers foster students' self-confidence and independence. (Sax et al. 2005) found that learners who maintain informal connections with their teachers gain stronger confidence both intellectually and emotionally. Similarly, (Schutz and DeCuir 2002) noted that empathic instructors enhance students' self-reliance in the school environment. Third, in terms of adaptability, friendly teacher-student interactions directly promote students' interpersonal adaptability. (Ampofo et al. 2025) further confirmed that teachers' empathy contributes to improved frustration tolerance among students.

For teachers with high agency, their leadership fosters a structured interactive environment, with positive implications for students' psychological *suzhi* across three core dimensions: First, in terms of cognitive traits, clear goal-setting and step-by-step guidance strengthen students' logical reasoning and metacognitive monitoring. Research on scaffolding instruction has demonstrated that structured guidance effectively enhances students' metacognitive capabilities (van de Pol et al., 2010). Second, regarding personality traits, appropriate strictness and rule-based guidance shape students' self-control, sense of responsibility, and independence. (Derakhshan et al. 2022) emphasized that responsible teaching practices are critical for boosting students' self-confidence, while (Sokol et al. 2015) noted that teachers' leadership plays a particularly important role in fostering students' creative attitudes. Third, in terms of adaptability, structured management helps students align with school rhythms and behavioral norms. Take structured teaching—characterized by explicitly communicated expectations, clear rules, and targeted guidance (Aelterman et al., 2019)—a body of research has demonstrated that this instructional approach contributes to improved student self-regulated learning abilities (Sierens et al., 2009; Mouratidis et al., 2013).

In addition, observational learning is also an important mechanism of internalization (Bandura, 2008). As mentioned earlier, teacher communication style is a stable personal characteristic of teachers. Through daily communication with class teachers, adolescents will, through active selection, acquire some of the teachers' styles and apply them in future interactions with others, thereby developing them into part of their own personal qualities (Zhang et al., 2013).

Psychological *suzhi* is an endogenous core factor of adaptive outcomes (Wang and Zhang, 2012a) and directly predicts individuals' adaptive states by exerting its functional value (Zhang et al., 2011). School adjustment, a key indicator for assessing mental health, is closely linked to the theoretical model proposed by (Wang and Zhang 2012a). (Zhang 2004) argued that the psychological *suzhi* of children is central to their mental structure and the essence of psychological processes, playing a dominant role. Mental health reflects the outward state of this structure and indicates the condition of psychological *suzhi*. Empirical research has shown that psychological *suzhi* is strongly correlated with various psychological and behavioral outcomes (Zhao et al., 2021b).

Research on psychological *suzhi* is regarded as an important component of positive psychology within the context of China's quality-oriented education (Zhang et al., 2017). Compared with psychological capital—a well-established Western construct in positive psychology—psychological *suzhi* holds unique value as a mediating variable in this study. Both belong to positive psychological resources yet differ fundamentally in essential attributes: psychological *suzhi* is a stable, trait-like quality formed through long-term environmental internalization, aligning with the stability of teachers' communication styles and well-suited to explaining long-term adjustment processes, whereas psychological capital is a dynamic, state-oriented resource (encompassing self-efficacy, hope, optimism, and resilience) that fluctuates with situational factors (Luthans et al., 2015; Zhang et al., 2017). Additionally, their structural compositions differ substantially: psychological *suzhi*'s three-dimensional framework directly corresponds to the multi-role influences of class teachers, while psychological capital—focused on state characteristics and limited to the aforementioned four dimensions—lacks independent cognitive and adaptability domains, preventing it from fully capturing the comprehensive impact of class teachers on adolescents' multi-dimensional school adjustment. Based on this, the following hypothesis is proposed:

*H3:* Psychological *Suzhi* mediates the associations between class teacher communication style (communion and agency) and adolescents' school adjustment.

### 1.4 Relationship between psychological *Suzhi* and basic psychological needs

There is an inherent and inevitable association between BPNs satisfaction and psychological *suzhi*, supported by two complementary theoretical frameworks. From the perspective of SDT (Ryan and Deci, 2017), the three BPNs serve as the core nourishment sources for human psychological development. Their satisfaction provides a direct foundation for the development of traits underlying psychological *suzhi*. In contrast, based on the cultural-historical theory, (Zhang et al. 2013) further revealed the underlying mechanism: psychological *suzhi* essentially emerges when individuals internalize external experiences into stable psychological structures through practical activities. Consequently, BPNs satisfaction serves as a crucial prerequisite for this internalization process, thereby offering process support for trait formation. Together, these two theories corroborate the inevitable association between the two constructs from the dual perspectives of trait development outcomes and internalization process mechanisms.

Specifically, the influence of each of the three needs on psychological *suzhi* manifests both in the direct nourishment of traits and the provision of critical support for the internalization process: (1)Autonomy satisfaction, on one hand, consistent with SDT, it fosters individuals' independence, initiative, and sense of responsibility (Ryan and Deci, 2017; Sierens et al., 2009), enhances self-discipline, and reduces procrastination (Tao and Jing, 2023), thereby directly optimizing the personality traits component of psychological *suzhi*; on the other hand, from the cultural-historical theory perspective, it transforms individuals' attitude toward external experiences from passive acceptance to proactive embrace; (2)Competence satisfaction provides positive feedback confirming experience effectiveness, strengthening individuals' willingness to transform external experiences into stable psychological qualities and offering motivational guarantees for the consistent development of psychological suzhi, with empirical studies showing that it enhances self-efficacy and self-confidence (Vansteenkiste et al., 2020), improves metacognitive functioning (Zhang et al., 2018), and promotes the development of self-regulated learning abilities (Mouratidis et al., 2013), thereby optimizing the cognitive dimensions and adaptive traits of psychological *suzhi*; (3)Relatedness satisfaction helps reduce individuals' defensive attitudes toward unfamiliar experiences, ensuring the quality of the internalization process, as research indicates that it enhances interpersonal coordination skills and reduces anxiety (Zhang et al., 2022), while positively predicting resilience-related traits such as grit (Çinar-Tanriverdi and Karabacak-Çelik, 2023; Du et al., 2023).

In summary, only when the three BPNs are satisfied to a certain extent can external experiences transcend situational experiences and evolve into stable components of psychological *suzhi*. This further confirms that the association between BPNs satisfaction and psychological *suzhi*.

This inherent association justifies the “BPNs satisfaction-psychological *suzhi*” serial mediating pathway between environmental variables and adaptive outcomes: Theoretically, environmental variables cannot directly affect long-term stable adaptive outcomes; they follow the classic sequence “environment → psychological process → stable quality → adaptive outcomes” (Lan et al., 2019; Lim et al., 2024). Specifically, environments first influence the dynamic process of BPNs (Ryan and Deci, 2017); continuous need satisfaction then drives external experiences to internalize into the stable psychological quality of psychological *suzhi*; finally, sound psychological *suzhi* directly acts on adaptive outcomes. In terms of variable attributes, BPNs satisfaction is a dynamic situational variable and cannot support long-term adaptive outcomes; in contrast, psychological *suzhi* is a stable implicit variable—a trait formed by long-term need satisfaction—which perfectly matches the long-term nature of adaptive outcomes.

Empirically, regarding the relationship between the two constructs (BPNs and Psychological *Suzhi*), direct empirical evidence for their association remains scarce—only (Liu et al. 2024) have revealed a moderate negative correlation BPNs frustration and psychological *suzhi*. However, psychological capital and psychological resilience, which are also classified as positive psychological resource variables alongside psychological *suzhi*, can provide further indirect empirical support for the relationship between BPNs and psychological *suzhi*. As we discussed earlier, we have elaborated on the differences between psychological *suzhi* and psychological capital in terms of their essential attributes and structural dimensions. However, in terms of their similarities, psychological *suzhi* and psychological capital exhibit consistency in both content and function. In terms of content composition, psychological capital is essentially embedded within psychological *suzhi*, with clear overlap in core qualities: self-efficacy corresponds to the self-confidence, rationality, and metacognitive monitoring/planning abilities of psychological *suzhi*; optimism aligns with the emotional adaptability, self-confidence, and rationality of psychological *suzhi*; hope links to the aspiration level and perseverance of psychological *suzhi*; and resilience matches the frustration tolerance of psychological *suzhi*. Functionally, both constructs contribute to individuals' adaptive functioning (Zhao et al., 2021b; Carmona-Halty et al., 2019). In addition, (Wang and Zhang 2012b) argued that psychological resilience is a subset of psychological *suzhi* in terms of content. Therefore, although psychological *suzhi* differs conceptually from psychological capital and psychological resilience, these constructs share a high degree of similarity.

Through a systematic review of studies examining the relationships between BPNs and these related constructs (i.e., psychological capital and psychological resilience), we found that BPNs exhibit significant predictive power for these variables. For example, (Verleysen et al. 2015) found that competence need plays a mediating role between appreciative inquiry and psychological capital. Similarly, (Carmona-Halty et al. 2019) demonstrated that psychological capital mediates the relationship between BPNs satisfaction and academic performance. In addition, (Liu and Huang 2021) proposed a human agency model, which suggests that resilience serves as a mediator between BPNs and individual outcome variables. Therefore, this study posits the following hypothesis:

*H4*: Basic psychological needs and psychological *suzhi* serially mediate the relationship between class teacher communication style (communion and agency) and adolescents' school adjustment.

In addition, psychological *suzhi* may also serve as a moderator in the mediating pathway of BPNs. According to the basic psychological need model, psychological qualities are considered potential moderators (Vansteenkiste et al., 2020). For example, (Liu et al. 2024) found that psychological *suzhi* moderated the effect of BPNs frustration on depression. (Wu et al. 2018) found that psychological *suzhi* moderated the association between bullying and social anxiety. In the study by (Chen et al. 2023), psychological *suzhi* moderated the effects of childhood maltreatment and inferiority complex on non-suicidal self-injury. (Pan and Zhang 2022) found that psychological *suzhi* moderated the relationship between stressful life events and college students' sleep quality. Likewise, (Zhao et al. 2021a) reported that psychological *suzhi* moderated the effect of school climate on adolescents' bullying victimization. Based on these findings, we propose the hypothesis:

*H5:* Psychological *suzhi* moderates the single mediation effect of basic psychological needs in the relationship between class teacher communication style (communion and agency) and adolescents' school adjustment.

**Figure 1:**
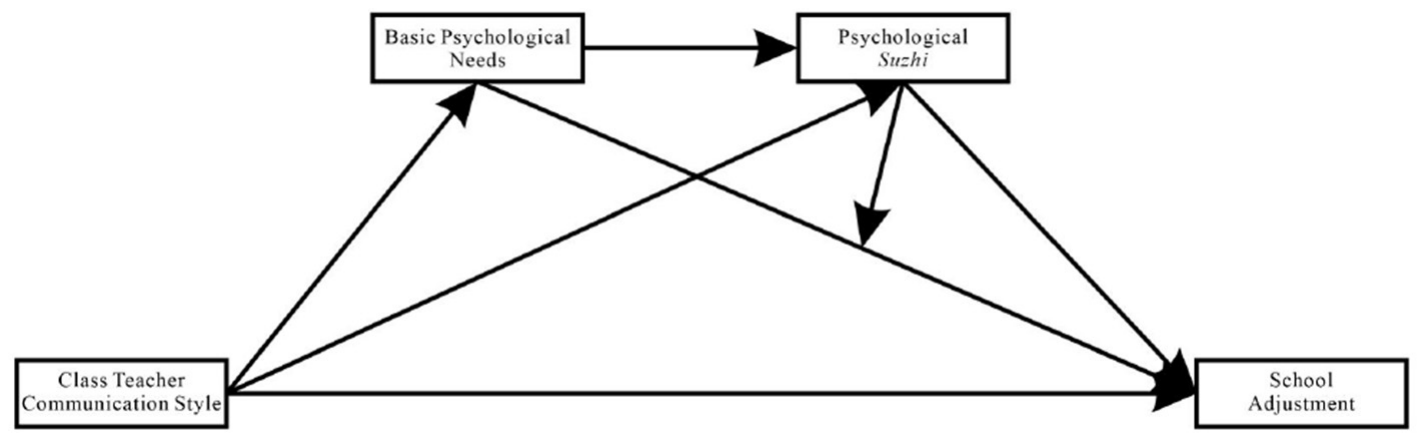
Theoretical hypothesis model conceptual diagram.

Taken together, the theoretical hypothesis model depicted in Figure 1 is proposed.

## 2 Method

### 2.1 Participants

This research administered a survey using questionnaires in 4 schools between May 2024 and June 2024. The schools included one urban secondary school, two rural schools (one junior high school and one senior high school), and one vocational technical school. The surveyed grades ranged from 7th grade to 11th grade. A total of 2,578 questionnaires were distributed. All 2,578 were returned. After cleaning the data, 160 invalid questionnaires were removed, resulting in an effective response rate of 93.79%. Our final sample included 51 classes, which is consistent with the recommendation of (Hox et al. 2017). They noted that at least 30–50 higher-level units are needed to ensure the reliability of data with a nested structure. The average number of students per class was 47, with a range of 18 to 65. This class size aligns with the typical range (10–30 students per class) suggested by (Hox et al. 2017) for ensuring sufficient within-class diversity, and the minimum class size of 18 further avoids potential bias from overly small groups. Overall, this sample structure provides a stable foundation for subsequent analyses of relationships between key variables.

The survey was carried out by graduate students with professional training, who guided the participants to complete the paper-and-pencil test during class time. The participants had one class period to finish the survey, with most completing it in 20–30 min. The studies involving human participants were reviewed and approved by the Ethics Committee of China West Normal University. A dual consent/assent process was implemented for this study. First, written consent was obtained from the guardian class directors at the school. Second, each adolescent participant received a written informed assent form, and research assistants verbally explained the study's purpose, content and core rights. The explanation explicitly stated that participation was voluntary and that adolescents could refuse to participate or withdraw at any time without negative consequences. Only after the adolescents signed the assent form did they proceed to complete the questionnaire.

### 2.2 Measures

#### 2.2.1 Class teacher communication styles

The Teacher Style Questionnaire, revised by (Jiang 2002) based on the U.S. version of the QTI developed by (Wubbels and Levy 1993), was used to assess students' perceptions of class teacher communication style. The questionnaire includes 64 items rated on a 5-point Likert scale (0 = never, 4 = always) and consists of eight subscales: leadership (DC), helpful/friendly (CD), understanding (CS), student freedom (SC), uncertain (SO), dissatisfied (OS), admonishing (OD), and strict (DO), with 8 items in each subscale. Each subscale score was calculated as the average of its corresponding 8 items. In the present study, McDonald's ω for the total scale was 0.96, with coefficients for the eight subscales ranging from 0.71 to 0.92.

To validate the two higher-order dimensions of the questionnaire—communion (cooperation–opposition) and agency (dominance–submission)—proposed by the Teacher Interpersonal Circle Model (Wubbels et al., 2014), a confirmatory factor analysis (CFA) was conducted using scores of the eight subscales. Results indicated good model fit (χ^2^ = 88.269, *df* = 8, *p* < 0.001; CFI = 0.995; TLI = 0.982; RMSEA = 0.064; SRMR = 0.028). Most subscales exhibited standardized factor loadings consistent with theoretical expectations, except for DO on the communion dimension: for communion, CD and CS had high positive loadings (0.805, 0.725), OS and OD showed high negative loadings (−0.845, −0.734), while DO showed a small positive loading (0.295) instead of the expected near-zero or negative one; for agency, DC and DO demonstrated positive loadings (0.302, 0.541), whereas SC and SO displayed moderate negative loadings (−0.310, −0.435). These results confirm the validity of the scale's two-dimensional structure in the present sample, thereby supporting the adoption of the theoretically derived weighting formulas (Den Brok et al., 2004)^1^ to compute communion and agency scores. Therefore, we derived the two higher-order dimension scores using these consistent weighting criteria.

#### 2.2.2 Basic psychological needs

The Chinese version of the Basic Psychological Need Satisfaction and Frustration Scale (BPNSFS), developed by (Chen et al. 2015), was used in this study. The scale includes 24 items that assess six dimensions: autonomy satisfaction, autonomy frustration, relatedness satisfaction, relatedness frustration, competence satisfaction, and competence frustration, with four items for each dimension. All items are rated on a 5-point Likert scale (1 = strongly disagree, 5 = strongly agree). Scores for autonomy, relatedness, and competence were derived by reverse-scoring the frustration items and averaging them with the corresponding satisfaction subscale scores. Higher scores indicate greater levels of need satisfaction. The McDonald's ω for the total scale was 0.89. The McDonald's ω values for the three subscales were 0.74 for autonomy, 0.79 for relatedness, and 0.83 for competence.

#### 2.2.3 Psychological Suzhi

Psychological *Suzhi* was measured using the Psychological *Suzhi* Questionnaire for Middle School Students (PSMQ), which was developed by (Hu et al. 2017). The questionnaire comprises 24 items, covering three subscales: cognitive traits (8 items; e.g., “I am very interested in new knowledge” and “When learning, I am good at connecting new knowledge with what I already know”), personality qualities (8 items; e.g., “I usually complete my tasks independently” and “I can face setbacks bravely without feeling disheartened or discouraged”), and adaptability (8 items; e.g., “I can often effectively defuse awkward situations” and “I can integrate into and adapt well to my current environment”). In this study, the McDonald's ω coefficient for the total scale was 0.93, and the McDonald's ω coefficients for the three subscales were 0.89, 0.83, and 0.82 for cognitive traits, personality qualities, and adaptability, respectively.

#### 2.2.4 School adjustment

Adolescent school adjustment was measured using the School Adjustment Questionnaire for Junior High School Students, developed by (Cui 2008). Many studies have demonstrated that the questionnaire has good reliability and validity among Chinese middle and high school students (Dong et al., 2023). The questionnaire consists of 27 items, covering five subscales: school attitude and emotions (7 items; e.g., “I dislike going to school”), peer relationships (6 items; e.g., “There is no one to play with me at school”), teacher-student relationships (5 items; e.g., “I am very scared of teachers”), academic adjustment (5 items; e.g., “I complete my homework very seriously”), and routine adjustment (4 items; e.g., “I often don't follow classroom rules”). The items are rated on a 5-point Likert scale (1 = strongly disagree, 5 = strongly agree), with some items reverse-scored. In this study, the McDonald's ω coefficient for the total scale was 0.93, and the McDonald's ω coefficients for the subscales ranged from 0.74 to 0.89.

### 2.3 Data analysis

Data analysis in this study was mainly conducted using Mplus 9.0 and SPSS 27.0. We used SPSS 27.0 to test the missing data pattern with Little's MCAR test. The result indicated that the data were not missing completely at random (χ*2*(42,604) = 50,362.893, *p* < 0.001). All item-level missing rates were below 1% (maximum = 0.79%). Therefore, we did not perform any imputation. After merging items (ignoring item-level missing values), there were no missing values for any variables.

Since this study relied entirely on self-report data, Harman's single-factor test was conducted via exploratory factor analysis to assess potential common method bias. Independent samples t-tests and one-way ANOVA were used to examine group differences in school adjustment, psychological *suzhi*, the three dimensions of BPNs, and the two dimensions of class teacher communication style across students' demographic characteristics. For descriptive statistics, we reported means, standard deviations, and intraclass correlation coefficients (ICCs) for communion, agency, autonomy, relatedness, competence, psychological *suzhi*, and school adjustment. Pearson correlation coefficients were used to test associations among class teacher communication style (communion and agency), three types of BPNs, psychological *suzhi*, and school adjustment.

In this study, the data exhibited a nested structure, with student-level scores (Level 1, L1) nested within class-level units (Level 2, L2). Results of the ICC further indicated that ICC values for class teacher communication style exceeded the threshold of 0.1 (communion: 0.27; agency: 0.36). It is generally recommended that a multilevel model should be employed for analysis when ICC values surpass this threshold (Marsh et al., 2012). At L2, the unit of analysis was the class, where variable scores were aggregated based on ratings provided by class members. The class-averaged score of teacher communication style reflects the more stable characteristics demonstrated by teachers in collective interaction contexts, corresponding to the individual-group interaction pattern. Its influence on adolescents' BPNs, psychological *suzhi*, and school adjustment is considered a “climate effect.” At L1, the unit of analysis was the individual, using student self-report data. The individual-level variance obtained by group-centering scores within classes can be interpreted as differences in students' perceptions of class teacher communication style during one-on-one interactions, corresponding to the individual-individual interaction pattern. Given the nested structure of the data, a multilevel model was adopted in this study to examine the total effect, mediating effect, and moderating effect.

We examined multicollinearity among the independent variables using Variance Inflation Factor (VIF) values. First, after controlling for gender, grade, boarding status, and subjective family economic status, we initially constructed Model 0 to examine the total effects of the two dimensions of class teacher communication style (at both L1 and L2) on adolescents' school adjustment.

Subsequently, we built multilevel multiple mediation models, where the two dimensions of class teacher communication style served as independent variables, BPNs as the first mediator, psychological *suzhi* as the second mediator, and school adjustment as the outcome variable. Given the distinct roles of the three BPNs, three separate mediation models (M1–M3) were constructed, with autonomy, relatedness, and competence respectively used as the indicator of BPNs in each model. Furthermore, considering the covariant characteristics among the three BPNs, we further developed a combined mediation model (M4) that included all three needs simultaneously to avoid “apparent effects.” These models did not incorporate moderation terms.

Building on these mediation models, we then added the interaction term between BPNs and psychological *suzhi* (at L1) to establish moderated multilevel multiple mediation models (M5–M8). We tested the single mediating effects of BPNs and psychological *suzhi*, as well as the serial mediation effect, at both L1 and L2. Additionally, we examined whether psychological *suzhi* (at L1) moderated the second half of the mediation path from teacher communication style to school adjustment via BPNs.

All indirect effects were tested using bias-corrected bootstrap confidence intervals. Analyses were conducted in Mplus 9.0. A 95% confidence interval (CI) that did not include zero was considered statistically significant. The significance level for all tests was set at *p* < 0.05.

## 3 Result

### 3.1 Common method bias test

The results indicated that, without rotation, 21 factors with eigenvalues exceeding 1 were identified, and the first factor accounted for 21.438% of the total variance, which is below the 40% threshold (Tang and Wen, 2020). As a result, no significant common method bias was found in this study.

### 3.2 Descriptive statistics and correlation analysis

Among the valid questionnaires, 1,246 students (51.5%) were male and 1,172(48.5%) were female. There were 425 students (17.6%) from 7th grade, 443(18.3%) from 8th grade, 413(17.1%) from 9th grade, 645(26.7%) from 10th grade, and 492(20.3%) from 11th grade. A total of 1,257 students (52.0%) were boarding students, and 1,161(48.0%) were day students. Regarding family residence, 1,090 students (45.1%) were from urban areas and 1,328(54.9%) from rural areas. For subjective family economic status, 113 students (4.7%) reported poor, 1,547(64.0%) average, 704(29.1%) good, and 39(1.6%) excellent, with 15 cases (0.6%) missing.

Table 1 presents the results of group comparisons on key variables (school adjustment, psychological *suzhi*, the three dimensions of BPNs, and the two dimensions of class teacher communication style) across demographic variables. Specifically, these comparisons revealed that gender, grade, boarding status, and subjective family economic status exhibited significant differences in most key variables (e.g., school adjustment, psychological *suzhi*), while family areas (urban vs. rural) showed significant differences only in psychological *suzhi* and the two dimensions of class teacher communication style. Therefore, gender, grade, boarding status, and subjective family economic status were included as control variables in subsequent analyses.

**Table 1:** Participants' characteristics and group differences in key study variables.

**Grouping variable**	**Outcome variable**	**t/F**	***P***	**Brief interpretation**
Gender	School adjustment	−3.630	< 0.001	2 > 1
	Psychological Suzhi	−6.616	< 0.001	2 > 1
	Autonomy	−3.568	< 0.001	2 > 1
	Relatedness	−0.943	0.346	—
	Competence	−11.672	< 0.001	2 > 1
	Agency	−0.273	0.785	—
	Communion	0.069	0.945	-
Grade	School Adjustment	16.643	< 0.001	2 = 1 > 3 = 4 > 5
	Psychological Suzhi	7.542	< 0.001	2 = 1 > 4 = 3 = 5
	Autonomy	12.359	< 0.001	2 > 3 = 1 = 4 > 5
	Relatedness	5.362	< 0.001	2 > 4 = 1 = 3 = 5, 4 > 5
	Competence	3.720	0.005	2 > 1 = 3 = 4 = 5
	Agency	25.393	< 0.001	1 > 2 = 4 > 5 = 3
	Communion	13.091	< 0.001	2 > 3 = 4 = 1 = 5, 3 = 4 > 5
Boarding Status	School Adjustment	−6.226	< 0.001	2 > 1
	Psychological Suzhi	−4.388	< 0.001	2 > 1
	Autonomy	−2.267	0.023	2 > 1
	Relatedness	−3.387	0.001	2 > 1
	Competence	−4.339	< 0.001	2 > 1
	Agency	−7.380	< 0.001	2 > 1
	Communion	−3.028	0.002	2 > 1
Family Areas	School adjustment	−1.411	0.158	—
	Psychological Suzhi	2.817	0.005	1 > 2
	Autonomy	0.579	0.562	—
	Relatedness	1.26	0.208	—
	Competence	1.436	0.151	—
	Agency	2.597	0.009	1 > 2
	Communion	−4.412	< 0.001	2 > 1
Subjective Family Economic Status	School Adjustment	28.136	< 0.001	3 = 4 > 2 > 1
	Psychological Suzhi	39.505	< 0.001	4 = 3 > 2 > 1
	Autonomy	31.636	< 0.001	4 > 3 > 2 > 1
	Relatedness	25.333	< 0.001	4 = 3 > 2 > 1
	Competence	29.634	< 0.001	4 > 3 > 2 = 1
	Agency	3.572	0.014	3 = 2 > 1 = 4
	Communion	6.573	< 0.001	3 > 2 > 1 = 4

Analytical methods: Independent samples *t*-test for binary variables; one-way ANOVA (LSD *post-hoc* test for significant groups) for multi-category variables. Group definitions: Gender (1, Female; 2, Male); Grade (1, Grade 7; 2, Grade 8; 3, Grade 9; 4, Grade 10; 5, Grade 11); Boarding Status (1, Boarding; 2, Day); Family Areas (1, Urban; 2, Rural); Subjective Family Economic Status (1, Poor; 2, Average; 3, Good; 4, Excellent). “2 > 1”, Group 2 > Group 1; “2 = 1 >3”, Group 2 = Group 1 > Group 3. Significance: *p* < 0.05.

Table 2 presents the means, standard deviations, ICCs and Pearson correlation coefficients for all study variables. The communion dimension of class teacher communication style was significantly positively correlated with autonomy (*r* = 0.376, *p* < 0.001), relatedness (*r* = 0.292, *p* < 0.001), and competence (*r* = 0.269, *p* < 0.001). It was positively correlated with psychological *suzhi* (*r* = 0.300, *p* < .001) and school adjustment (*r* = 0.447, *p* < 0.001). The agency dimension of class teacher communication style was significantly positively correlated with relatedness (*r* = 0.110, *p* < 0.001), competence (*r* = 0.064, *p* = 0.002), and positively correlated with psychological *suzhi* (*r* = 0.131, *p* < 0.001) and school adjustment (*r* = 0.102, *p* < 0.001). All three types of BPNs were significantly positively correlated with psychological *suzhi* (*r* = 0.532, 0.496, 0.654; all *p* < 0.001, respectively) and with school adjustment (*r* = 0.590, 0.566, 0.595; all *p* < 0.001, respectively). Psychological *suzhi* was strongly positively correlated with school adjustment (*r* = 0.673, *p* < 0.001).

**Table 2:** Descriptive statistics, correlation analysis.

**No**.	**Variable name**	**M ±SD**	**Range**	**ICC**	**1**	**2**	**3**	**4**	**5**	**6**
1	Communion	3.31 ± 3.35	−10.4–10.4	0.271	1					
2	Agency	2.02 ± 1.51	−10.4–10.4	0.358	−0.149^***^	1				
3	Autonomy	3.40 ± 0.67	1–5	0.039	0.376^***^	0.029	1			
4	Relatedness	3.67 ± 0.73	1–5	0.036	0.292^***^	0.110^***^	0.564^***^	1		
5	Competence	3.20 ± 0.79	1–5	0.041	0.269^***^	0.064^**^	0.631^***^	0.536^***^	1	
6	Psychological Suzhi	3.15 ± 0.62	1–5	0.070	0.300^***^	0.131^***^	0.532^***^	0.496^***^	0.654^***^	1
7	School Adjustment	3.72 ± 0.68	1–5	0.074	0.447^***^	0.102^***^	0.590^***^	0.566^***^	0.595^***^	0.673^***^

^*^p < 0.05.

^**^p < 0.01.

^***^p < 0.001.

M, mean; SD, standard deviation; ICC, Intraclass correlation coefficient.

### 3.3 Moderated mediation effect analysis

The VIF test results showed that, in all models, the highest VIF values for all predictor variables (including control variables) ranged from 1.324 to 2.345, all well below the commonly accepted threshold of 5. Therefore, it can be concluded that multicollinearity was not a concern in this study (O'brien, 2007).

Beyond the control variables, all L1 variables were group-mean centered. Interaction terms were then created by multiplying autonomy, relatedness, and competence with psychological *suzhi*, respectively. After controlling for gender, boarding status, and subjective family economic status at L1, and grade at L2, we first examined the total effects of class teacher communication style on adolescents' school adjustment. Results indicated that at L1: (1) communion significantly and positively predicted school adjustment (β = 0.473, *p* < 0.001); and (2) agency also significantly and positively predicted school adjustment (β = 0.103, *p* < 0.001). At L2, the pattern was consistent: (1) communion significantly and positively predicted school adjustment (β = 0.488, *p* < 0.001); and (2) agency also significantly and positively predicted school adjustment (β = 0.398, *p* < 0.001).

**Figure 2:**
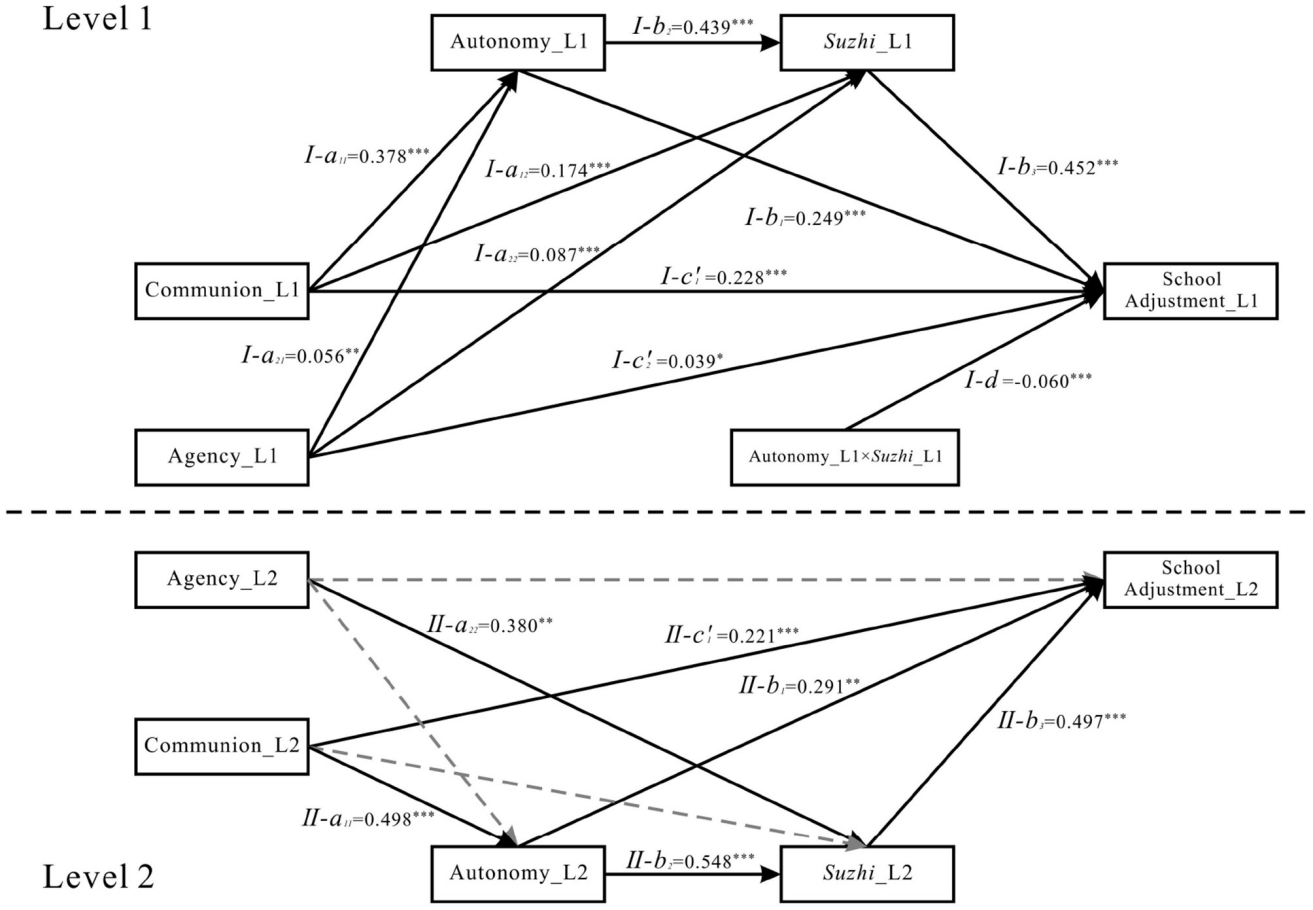
Model M5 statistical diagram. *Suzhi*, psychological *suzhi*. ***p < 0.001, **p < 0.01 and *p < 0.05.

**Figure 3:**
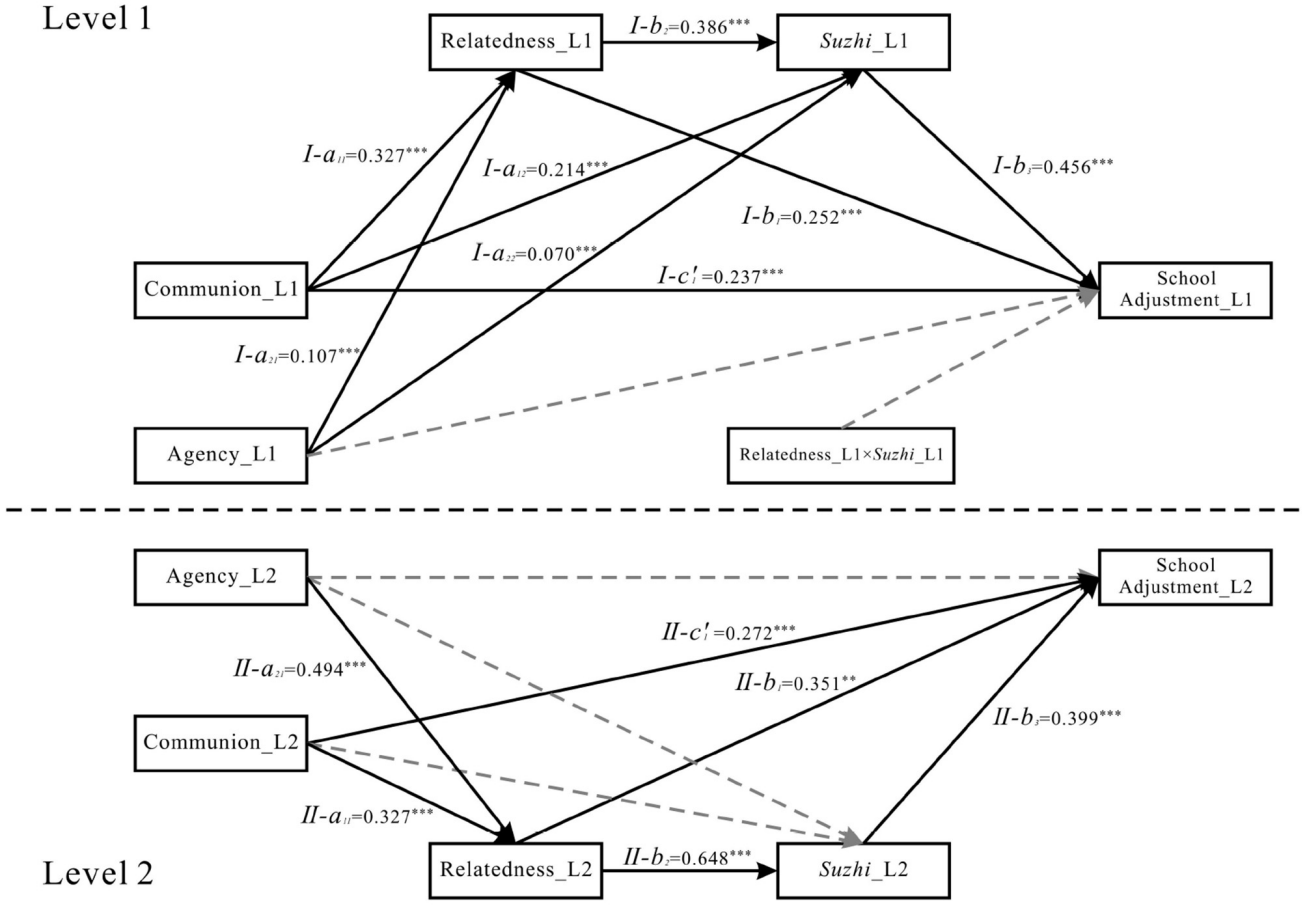
Model M6 statistical diagram. *Suzhi*, psychological *suzhi*. Dashed lines indicate non-significant paths. ***p < 0.001, **p < 0.01 and *p < 0.05.

**Figure 4:**
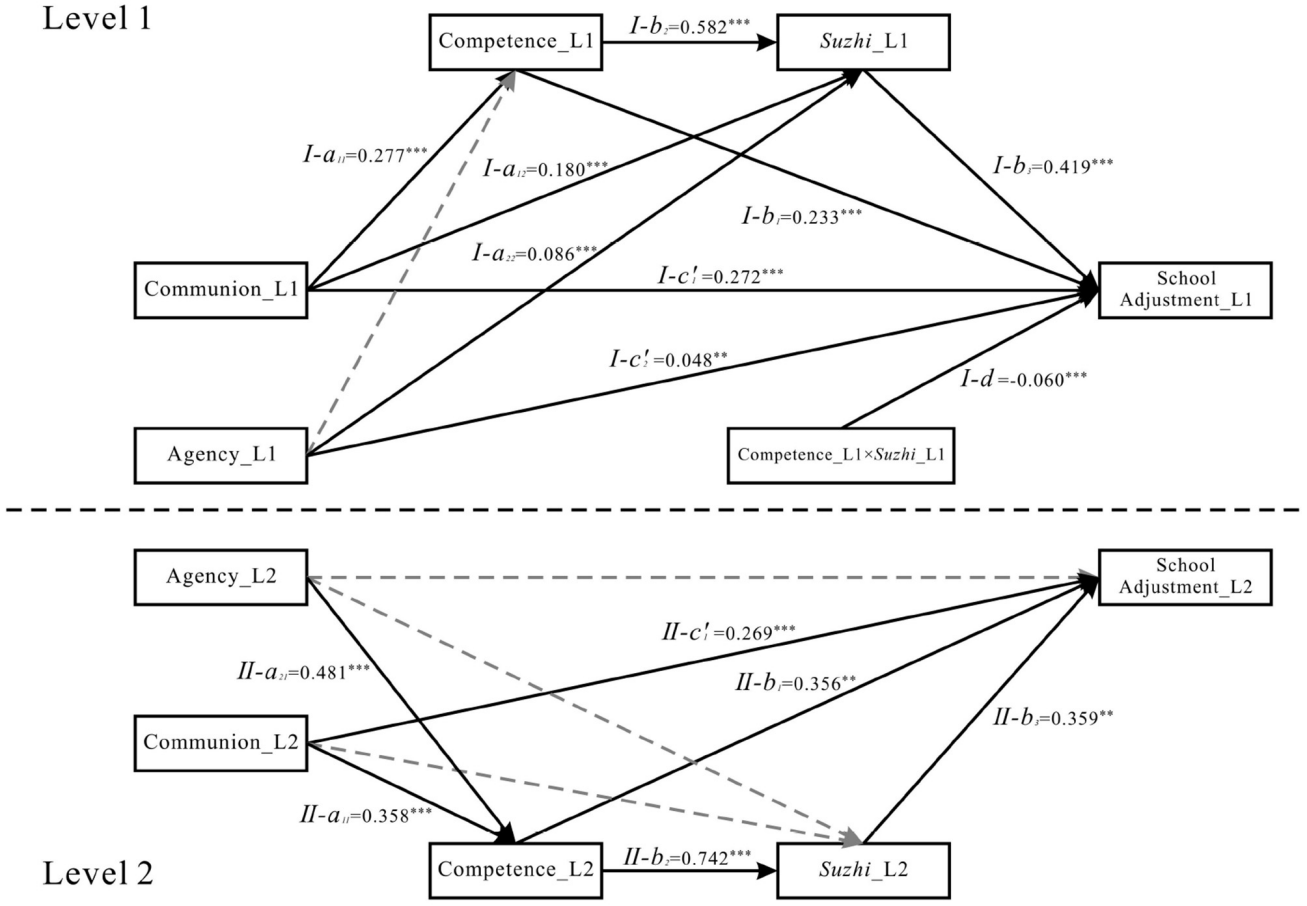
Model M7 statistical diagram. *Suzhi*, psychological *suzhi*. Dashed lines indicate non-significant paths. ***p < 0.001, **p < 0.01 and *p < 0.05.

**Figure 5:**
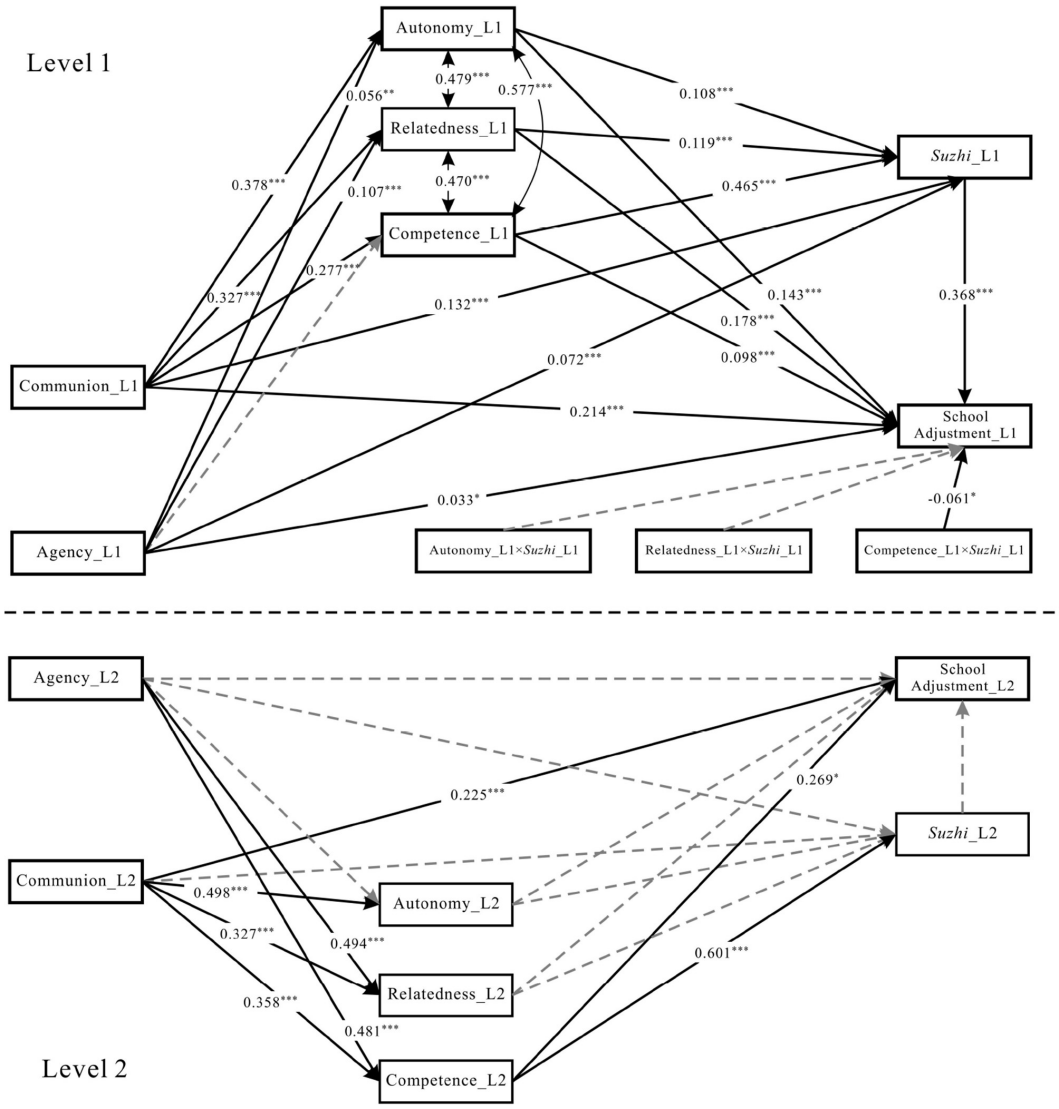
Model M8 statistical diagram. *Suzhi*, Psychological *suzhi*. Dashed lines indicate non-significant paths. ***p < 0.001, **p < 0.01 and *p < 0.05.

Subsequently, we tested the multilevel multiple mediation models (M1–M4, without interaction terms). Results revealed that at L1 of M1–M4, all mediating effects of the three BPNs between class teacher communication style and school adjustment were significant, except that competence did not mediate the effect of agency on school adjustment in M3 and M4. This finding laid the foundation for testing the moderating effects in M5–M8. We then examined the moderated multilevel mediation models (M5–M8) by incorporating the interaction terms. For brevity, only the results of M5–M8 are reported herein, with the path coefficients of these models presented in Figures 2–5, respectively.

Mediation effects were tested using the bias-corrected bootstrap method with 5,000 resamples. As shown in Table 3 (M5), Table 4 (M6), and Table 5 (M7), the three separate need models revealed level-specific mediation patterns across both L1 and L2. For the communion-school adjustment relationship, all three BPNs exhibited significant single mediation at both levels, while their serial mediation with psychological *suzhi* also maintained significance across L1 and L2. For the agency-school adjustment relationship, BPNs mediation showed clearer level differentiation: autonomy mediated significantly only at L1, relatedness at both levels, and competence solely at L2—patterns consistent for both single and serial mediation with psychological *suzhi*. Psychological *suzhi*'s single mediation followed distinct rules: it significantly mediated communion-school adjustment only at L1, and agency-school adjustment at L1 across all models, with L2 significance limited to the autonomy-focused model (M5) alone.

**Table 3:** Mediating effects of autonomy and psychological *Suzhi* (model M5).

**Model**	**Level**	**Independent variable**	**Path**	**Effect**	**Boot SE**	**Boot LLCI**	**Boot ULCI**	**Relative effect**
M5	L1	Communion	Total effect	0.476	0.024	0.428	0.522	100.00%
			Direct effect	0.228	0.020	0.188	0.268	47.90%
			Single mediation effect (A)	0.094	0.009	0.078	0.112	19.75%
			Single mediation effect (Suzhi)	0.079	0.009	0.061	0.097	16.60%
			Serial mediation effect (A-Suzhi)	0.075	0.006	0.064	0.087	15.76%
		Agency	Total effect	0.103	0.020	0.064	0.143	100.00%
			Direct effect	0.039	0.016	0.007	0.069	37.86%
			Single mediation effect (A)	0.014	0.005	0.005	0.023	13.59%
			Single mediation effect (Suzhi)	0.039	0.007	0.025	0.053	37.86%
			Serial mediation effect (A-Suzhi)	0.011	0.004	0.004	0.018	10.68%
	L2	Communion	Total effect	0.488	0.066	0.289	0.765	100.00%
			Direct effect	0.221	0.050	0.200	0.380	45.29%
			Single mediation effect (A)	0.145	0.040	0.111	0.312	29.71%
			Single mediation effect (Suzhi)	−0.009	0.036	−0.070	0.074	−1.84%
			Serial mediation effect (A-Suzhi)	0.131	0.031	0.112	0.266	26.84%
		Agency	Total effect	0.398	0.133	0.118	0.701	100.00%
			Direct effect	0.121	0.113	−0.123	0.314	30.40%
			Single mediation effect (A)	0.050	0.051	−0.049	0.152	12.56%
			Single mediation effect (Suzhi)	0.182	0.083	0.052	0.387	45.73%
			Serial mediation effect (A-Suzhi)	0.045	0.045	−0.033	0.138	11.31%

A, autonomy; *Suzhi*, Psychological *suzhi*.

**Table 4:** Mediating effects of relatedness and psychological *Suzhi* (model M6).

**Level**	**Independent variable**	**Path**	**Effect**	**Boot SE**	**Boot LLCI**	**Boot ULCI**	**Relative effect**
L1	Communion	Total effect	0.474	0.024	0.425	0.520	100.00%
		Direct effect	0.237	0.020	0.198	0.278	50.00%
		Single mediation effect (R)	0.082	0.009	0.067	0.100	17.30%
		Single mediation effect (Suzhi)	0.098	0.010	0.079	0.119	20.68%
		Serial mediation effect (R-Suzhi)	0.058	0.005	0.048	0.067	12.24%
	Agency	Total effect	0.103	0.020	0.065	0.143	100.00%
		Direct effect	0.025	0.016	−0.006	0.056	24.27%
		Single mediation effect (R)	0.027	0.005	0.018	0.036	26.21%
		Single mediation effect (Suzhi)	0.032	0.007	0.019	0.045	31.07%
		Serial mediation effect (R-Suzhi)	0.019	0.003	0.013	0.026	18.45%
L2	Communion	Total effect	0.488	0.066	0.289	0.764	100.00%
		Direct effect	0.272	0.043	0.136	0.407	55.74%
		Single mediation effect (R)	0.115	0.043	0.066	0.270	23.57%
		Single mediation effect (Suzhi)	0.017	0.019	−0.008	0.074	3.48%
		Serial mediation effect (R-Suzhi)	0.085	0.029	0.057	0.202	17.42%
	Agency	Total effect	0.398	0.133	0.117	0.627	100.00%
		Direct effect	0.036	0.105	−0.181	0.225	9.05%
		Single mediation effect (R)	0.173	0.077	0.065	0.345	43.47%
		Single mediation effect (Suzhi)	0.062	0.062	−0.066	0.173	15.58%
		Serial mediation effect (R-Suzhi)	0.128	0.056	0.024	0.216	32.16%

R, relatedness; *Suzhi*, psychological *suzhi*.

**Table 5:** Mediating effects of competence and psychological *Suzhi* (model M7).

**Level**	**Independent variable**	**Path**	**Effect**	**Boot SE**	**Boot LLCI**	**Boot ULCI**	**Relative effect**
L1	Communion	Total effect	0.479	0.024	0.431	0.524	100.00%
		Direct effect	0.272	0.021	0.232	0.313	56.78%
		Single mediation effect (C)	0.065	0.008	0.051	0.080	13.57%
		Single mediation effect (Suzhi)	0.075	0.009	0.060	0.093	15.66%
		Serial mediation effect (C-Suzhi)	0.067	0.006	0.055	0.080	13.99%
	Agency	Total effect	0.105	0.020	0.068	0.145	100.00%
		Direct effect	0.048	0.015	0.020	0.078	45.71%
		Single mediation effect (C)	0.010	0.006	0.000	0.022	9.52%
		Single mediation effect (Suzhi)	0.036	0.007	0.022	0.050	34.29%
		Serial mediation effect (C-Suzhi)	0.011	0.005	0.000	0.021	10.48%
L2	Communion	Total effect	0.488	0.066	0.289	0.764	100.00%
		Direct effect	0.269	0.045	0.136	0.408	55.12%
		Single mediation effect (C)	0.128	0.039	0.091	0.364	26.23%
		Single mediation effect (Suzhi)	−0.004	0.021	−0.061	0.026	−0.82%
		Serial mediation effect (C-Suzhi)	0.096	0.031	0.067	0.249	19.67%
	Agency	Total effect	0.398	0.133	0.118	0.627	100.00%
		Direct effect	0.057	0.104	−0.165	0.246	14.32%
		Single mediation effect (C)	0.171	0.079	0.056	0.344	42.96%
		Single mediation effect (Suzhi)	0.042	0.051	−0.048	0.154	10.55%
		Serial mediation effect (C-Suzhi)	0.128	0.069	0.035	0.278	32.16%

C, competence; *Suzhi*, psychological *suzhi*.

As presented in Table 6, comparisons between individual models (M5–M7) and the combined need model (M8) identified limited discrepancies, all confined to L2. Specifically, psychological *suzhi*'s single mediation of agency-school adjustment, significant at L2 only in M5 among individual models, became non-significant in M8; the serial pathways “communion → autonomy → psychological *suzhi* → school adjustment” and “agency → relatedness → psychological *suzhi* → school adjustment,” significant in their respective individual models, lost significance in M8. Additionally, effect sizes for all corresponding mediating pathways in M8 were consistently smaller than those in individual models.

**Table 6:** Mediating effects of basic psychological needs and psychological *Suzhi* (combined model M8).

**Level**	**Independent variable**	**Path**	**Effect**	**Boot SE**	**Boot LLCI**	**Boot ULCI**	**Relative effect**
L1	Communion	Total effect	0.479	0.024	0.431	0.524	100.00%
		Direct effect	0.214	0.019	0.176	0.252	44.68%
		Single mediation effect (A)	0.054	0.008	0.038	0.070	11.27%
		Single mediation effect (R)	0.058	0.008	0.044	0.075	12.11%
		Single mediation effect (C)	0.027	0.007	0.014	0.043	5.64%
		Single mediation effect (Suzhi)	0.049	0.007	0.036	0.065	10.23%
		Serial mediation effect (A-Suzhi)	0.015	0.004	0.007	0.023	3.13%
		Serial mediation effect (R-Suzhi)	0.014	0.002	0.010	0.019	2.92%
		Serial mediation effect (C-Suzhi)	0.047	0.005	0.038	0.058	9.81%
	Agency	Total effect	0.105	0.020	0.068	0.146	100.00%
		Direct effect	0.033	0.015	0.005	0.062	31.43%
		Single mediation effect (A)	0.008	0.003	0.003	0.014	7.62%
		Single mediation effect (R)	0.019	0.004	0.013	0.027	18.10%
		Single mediation effect (C)	0.004	0.003	0.000	0.011	3.81%
		Single mediation effect (Suzhi)	0.027	0.006	0.015	0.038	25.71%
		Serial mediation effect (A-Suzhi)	0.002	0.001	0.001	0.004	1.90%
		Serial mediation effect (R-Suzhi)	0.005	0.001	0.003	0.007	4.76%
		Serial mediation effect (C-Suzhi)	0.007	0.004	0.000	0.015	6.67%
L2	Communion	Total effect	0.534	0.072	0.289	0.764	100.00%
		Direct effect	0.225	0.053	0.205	0.335	42.13%
		Single mediation effect (A)	0.063	0.043	0.007	0.208	11.80%
		Single mediation effect (R)	0.085	0.041	0.034	0.243	15.92%
		Single mediation effect (C)	0.096	0.038	0.059	0.295	17.98%
		Single mediation effect (Suzhi)	−0.012	0.018	−0.088	0.008	−2.25%
		Serial mediation effect (A-Suzhi)	0.000	0.015	−0.027	0.039	0.00%
		Serial mediation effect (R-Suzhi)	0.026	0.015	0.010	0.103	4.87%
		Serial mediation effect (C-Suzhi)	0.051	0.023	0.028	0.289	9.55%
	Agency	Total effect	0.436	0.139	0.118	0.627	100.00%
		Direct effect	0.036	0.124	−0.225	0.267	8.26%
		Single mediation effect (A)	0.022	0.036	−0.030	0.121	5.05%
		Single mediation effect (R)	0.128	0.090	0.004	0.287	29.36%
		Single mediation effect (C)	0.129	0.081	0.013	0.271	29.59%
		Single mediation effect (Suzhi)	0.014	0.043	−0.052	0.122	3.21%
		Serial mediation effect (A-Suzhi)	0.000	0.012	−0.023	0.031	0.00%
		Serial mediation effect (R-Suzhi)	0.039	0.039	−0.019	0.128	8.94%
		Serial mediation effect (C-Suzhi)	0.068	0.050	0.004	0.165	15.60%

A, autonomy; R, relatedness; C, competence; *Suzhi*, psychological *suzhi*.

For the moderated mediation analyses, we focused on psychological *suzhi*'s moderating role in the BPNs-to-school adjustment link at L1, with results compared across individual need models (M5–M7) and the combined model (M8).

For the autonomy-focused moderating pathway: The interaction between autonomy and psychological *suzhi* significantly predicted school adjustment in M5 (β = −0.060, *p* < 0.001; see Figure 2). The bootstrap 95% CIs for the product of the path coefficient from communion to autonomy (*I-a*_11_) and the interaction term (*I-d*), as well as the product of the path coefficient from agency to autonomy (*I-a*_21_) and the interaction term (*I-d*)—unstandardized, as Mplus 9.0 cannot output standardized CIs for products of coefficients in New/Additional Parameters—both excluded zero [−0.011, −0.004; −0.005, −0.001], confirming a significant moderating effect. However, this moderating effect became non-significant in M8 (β = −0.026, *p* = 0.342; see Figure 5), indicating its lack of stability when controlling for the covariances among BPN dimensions.

For the relatedness-focused moderating pathway: The interaction between relatedness and psychological *suzhi* did not significantly predict school adjustment in either M6 (β = −0.023, *p* = 0.218; see Figure 3) or M8 (β = 0.022, *p* = 0.298; see Figure 5), indicating no significant moderating effect of psychological *suzhi* on this pathway.

For the competence-focused moderating pathway: The interaction between competence and psychological *suzhi* significantly predicted school adjustment in both M7 (β = −0.060, *p* < 0.001; see Figure 4) and M8 (β = −0.061, *p* = 0.022; see Figure 5), showing stable cross-model significance. The bootstrap 95% CIs for the product of the path coefficient from communion to competence (*I-a*_11_) and the interaction term (*I-d*) —unstandardized, as noted above—excluded zero in both models [−0.008, −0.002 for M7; −0.010, −0.001 for M8], confirming that psychological *suzhi* stably moderated this mediation pathway. However, the product of the path coefficient from agency to competence (*I-a*_21_) and the interaction term (*I-d*) yielded 95% CIs that included zero [−0.005, 0.000 for M7; −0.006, 0.000 for M8], indicating that the moderating effect on this mediation pathway was non-significant.

**Figure 6:**
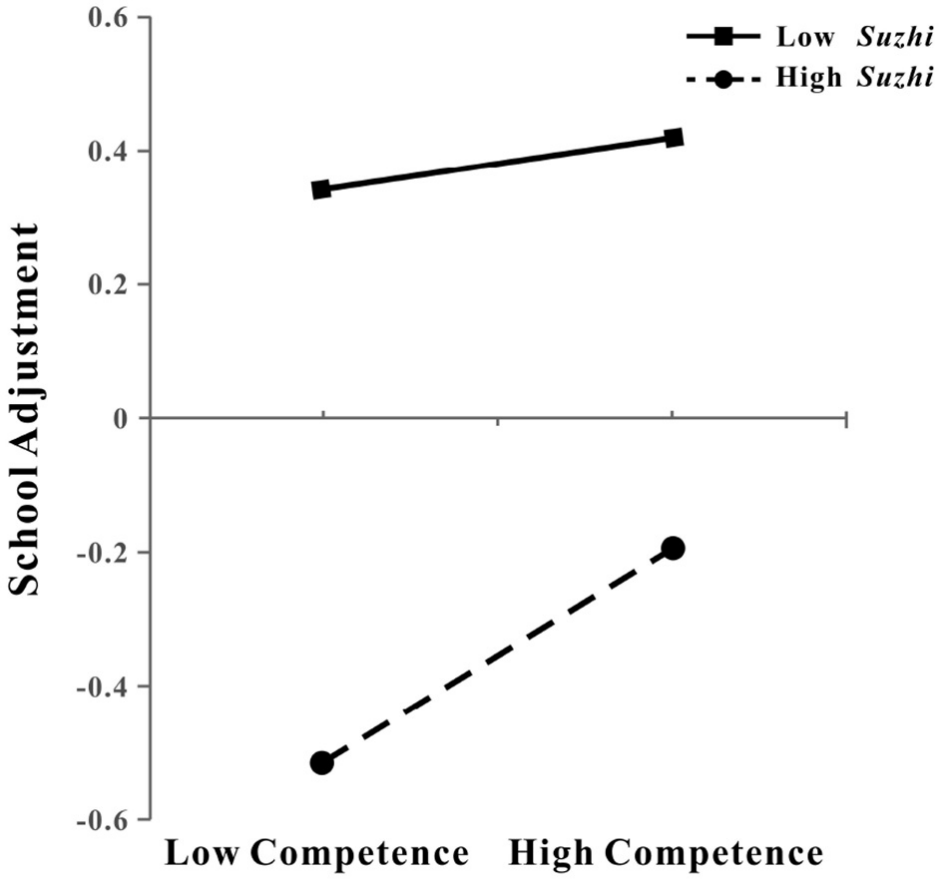
The moderating effect of psychological *Suzhi* on the relationship between competence and adolescents' school adjustment. *Suzhi*, psychological *suzhi*.

A simple slope analysis was conducted focusing on competence (based on the combined model M8), given its stable moderating effect across individual and combined models. Results showed that when adolescents had low levels of psychological *suzhi* (M – 1SD), competence significantly and positively predicted school adjustment (β_simple_ = 0.159, *t* = 4.543, *p* < 0.001; see Figure 6). In contrast, when adolescents had high levels of psychological *suzhi* (M + 1SD), the positive predictive effect of competence on school adjustment became non-significant (β_simple_ = 0.037, *t* = 0.961, *p* = 0.337; see Figure 6), with a notably less steep slope indicating that the predictive role of competence diminished under high psychological *suzhi*.

## 4 Discussion

This study aimed to examine the effects of two dimensions of class teacher communication style on Chinese adolescents' school adjustment, based on student self-reported data. It further investigated the mediating roles of three BPNs as well as psychological *suzhi*. In addition, the moderating effect of psychological *suzhi* on these relationships was explored. A moderated multilevel multiple mediation model was proposed and tested.

### 4.1 Class teacher communication style and school adjustment

The findings supported hypothesis H1. Both dimensions of class teacher communication style had significant positive effects on adolescents' school adjustment at both levels. This result is consistent with the core assumption of ecological systems theory, which highlights the critical role of teachers as proximal agents in the school microsystem (Bronfenbrenner, 1992). Through daily interactions, class teachers shape adolescents' psychological development and adjustment to the school environment.

Specifically, the communion dimension positively predicted school adjustment. Compared to a hostile communication style, a friendly and cooperative communication style better supports students' emotional security and sense of trust (Ruby, 2025). According to attachment theory, the teacher–student relationship can serve as a “secondary attachment bond” (Pianta, 1999; García-Rodríguez et al., 2023). When teachers demonstrate warmth and cooperation, students are more likely to develop a secure attachment, which facilitates their adjustment. In contrast, a hostile style may foster insecure attachment, leading to defensive or antagonistic responses that hinder adjustment.

The agency dimension also significantly predicted school adjustment. A dominant communication style, compared to a submissive one, was more beneficial to students' adaptation. This finding supports authoritative leadership theory, which emphasizes that structured, directive teacher behaviors can promote students' adjustment in school settings (Wentzel, 2002).

In comparing the relative strengths of the two dimensions, the effect of communion was greater than that of agency at both the L1 and L2. This pattern aligns with previous research and interpersonal theory, emphasizing the central role of emotional support in adolescent school adjustment (Jiang, 2002). When examining the effects across levels, the magnitude of communion's effect showed little difference between L1 and L2; however, agency's effect was stronger at L2 than at L1. A possible explanation for this is that class teacher's agency is primarily manifested through collective management approaches such as setting class rules and shaping class culture. Therefore, it is logical that the effect of agency is stronger at L2 than at L1.

### 4.2 The mediating role of basic psychological needs

The results supported Hypothesis H2. Specifically, at the L1 level, each of the BPNs independently mediated the relationship between the two dimensions of class teachers' communication styles and adolescents' school adjustment, except that competence did not significantly mediate the effect of agency on school adjustment alone (this path was significant at the L2). Furthermore, this pattern was consistent across both the combined model and separate models. This finding provides new empirical support for the basic psychological need model and further highlights that class teachers' communication styles—stable personal characteristics of teachers—serve as a critical contextual factor influencing students' psychological need satisfaction.

Specifically, along the communion dimension, class teachers' friendly and cooperative communication style can be interpreted as a form of relatedness-supportive behavior characterized by care and warmth (Ryan and Deci, 2017). Such behavior facilitates the satisfaction of students' BPNs, thereby promoting better school adjustment. Meanwhile, students' long-term interactions with their class teachers across multiple contexts shape stable expectations of teachers' behaviors, thereby facilitating their psychological need satisfaction and enhancing their level of school adjustment (Lemay and Venaglia, 2016).

In the agency dimension, results showed that a dominant communication style was more conducive to satisfying students' needs for autonomy (L1), relatedness (L1 and L2), and competence (L2). This pattern aligns with prior observations that dominant teachers often set higher expectations and offer clearer goals—thereby fostering a classroom climate focused on effort and achievement. Such behavior has been considered competence-supportive (Standage et al., 2005; Patzak and Zhang, 2025), as it can help students feel more capable. A further contribution of the present study is the finding that this effect is more strongly explained by between-class variance, suggesting that the influence of teachers' communication styles on adolescents' competence need satisfaction may be primarily realized through collective management approaches.

However, the positive effects of agency on autonomy and relatedness appear somewhat counterintuitive. Unlike its effect on competence, theoretically, dominance does not seem to align with the characteristics of a traditionally autonomy-supportive or relatedness-supportive environment, while submission is often viewed as offering students greater opportunities for self-determination and relational closeness. Yet, in this study, students reported higher levels of autonomy and relatedness under dominant teachers, whereas these needs were not significantly enhanced under submissive ones.

This seemingly paradoxical result can be interpreted from two perspectives. First, drawing on SDT, researchers have distinguished between two types of high-directiveness teacher styles: structure, and control. According to (Aelterman et al. 2019), control is need-thwarting, whereas structure is need-supportive. A dominant style, when not controlling, may reflect structured guidance provided by the teacher. For adolescents in the process of constructing their self-identity, such structure can help reduce confusion, provide direction, and foster a sense of volitional functioning, thereby increasing autonomy satisfaction.

Second, this phenomenon may be rooted in the Chinese cultural context, where teachers are traditionally regarded as authoritative figures expected to “impart knowledge and resolve doubts.” Within this cultural framework, dominance may be interpreted as a sign of responsibility and competence, whereas submissiveness could be viewed as a lack of authority or even negligence. Consequently, students may respond more positively—both emotionally and motivationally—to teachers who exhibit a dominant communication style.

Taken together, these findings suggest that autonomy support should not be equated with submission (Aelterman et al., 2019; Patzak and Zhang, 2025). A consistently submissive approach may fail to provide students with the clear structure they need to navigate their development, potentially resulting in uncertainty and frustration that undermine need satisfaction. Particularly in the Chinese educational context, teachers should adopt a structured and guiding form of dominance to support student growth. At the same time, care must be taken to ensure that such dominance does not become controlling. Consistent with this view, (Jang et al. 2010) have also emphasized that the optimal strategy for promoting adolescents' school adjustment combines structured authority with autonomy support.

However, caution is warranted regarding whether the predictive effect of the agency dimension on autonomy and relatedness needs is culturally bounded—in other words, this effect may reverse or even vanish in mainstream Western educational contexts. In existing studies conducted in Western cultural contexts, the construct most closely aligned with the agency dimension of teacher communication style is teacher structural support; meta-analyses and reviews have shown that structural support is not opposed to autonomy support but instead collaborates with it to enhance students' perceived autonomy and competence (Okada, 2021; Patzak and Zhang, 2025), suggesting that teachers high in the agency dimension may similarly positively predict students' perceived autonomy need satisfaction in Western contexts. However, no empirical evidence linking structural support to relatedness need satisfaction has been identified.

It is important to clarify, as noted earlier, that teacher structural support is confined to instructional contexts, whereas teacher communication style constitutes teachers' cross-contextual stable interpersonal tendency. Furthermore, no empirical studies have measured teacher communication style using the QTI or explored its relationship with BPNs. Given this, we remain cautious about the generalizability of our conclusions to other cultural contexts.

Finally, it is important to note that although some independent mediating pathways of BPNs are statistically significant at L2, caution is required when interpreting these L2 BPN-mediated effects. This is because the highest ICC among the three BPNs dimensions is only 0.041—far below the commonly accepted threshold of 0.1 for multilevel model analysis (Hox et al., 2017)—even though the number of classes (51) in this study meets the minimum sample size requirement for multilevel analysis (30–50 higher-level units) proposed by (Hox et al. 2017).

Furthermore, a comparison between the separate need models (M5–M7) and the combined need model (M8) reveals that the statistical significance of the independent mediating pathways of each BPN dimension between class teacher communication style and school adjustment is entirely consistent across the two types of models. This consistency indicates that these mediating mechanisms are stable and reliable. Notably, however, the effect sizes of the mediating pathways in the combined model are significantly smaller than those in the separate models. This difference reminds readers that when interpreting the effect sizes of BPN-mediated effects, the results of the two model types should be analyzed together: the effect sizes in the combined model are obtained after controlling for the other two needs, reflecting the “net effect” after excluding overlapping variance among needs; in contrast, the mediating effect sizes of each BPN in the separate models represent the “total effect” that includes the joint contribution of different needs.

### 4.3 The mediating role of psychological *Suzhi*

The results support Hypothesis H3, indicating that at L1, psychological *suzhi* acts as a single mediator between class teachers' communication styles and adolescents' school adjustment. Prior research (Chen et al., 2018) emphasized that teacher support—emotional, competence-based, and academic—is a key external factor in the development of adolescents' psychological *suzhi*. The present findings extend this by showing that teachers' interpersonal characteristics, particularly communication style, also play a significant role. This provides new empirical support for the psychological *suzhi* development model proposed by (Wang and Zhang 2012a), which highlights the importance of environmental influences.

In the communion dimension, teachers' cooperative and friendly interactions were associated with greater psychological *suzhi*, which in turn promoted better school adjustment. (Cheng et al. 2025) found a moderate positive correlation between teacher emotional support and students' psychological resilience and emotional intelligence, suggesting that teachers with high communion traits may strengthen students' self-esteem, emotional stability and frustration tolerance, thereby fostering personality development.

In the agency dimension, a more dominant communication style similarly contributed to school adjustment via enhanced psychological *suzhi*. Adolescence is a critical stage for developing rule awareness, self-regulation, and cognitive control, during which students are especially susceptible to distractions. Teachers who demonstrate leadership and set clear expectations are more likely to establish stable and structured classroom environments (Jiang, 2002). Such environments foster cognitive traits such as attention control, self-monitoring, and problem-solving, as well as personality traits like responsibility and emotional regulation. By enhancing these dimensions of psychological *suzhi*, a dominant communication style supports students' resilience and overall adjustment.

Furthermore, from a hierarchical perspective, at L2, most of the single mediating pathways of psychological *suzhi* are non-significant. The ICC values of psychological suzhi do not exceed the 0.1 threshold, which implies that psychological *suzhi*—as a stable trait formed by individuals through long-term internalization of environmental stimuli—derives its variations mainly from individuals' active selection and processing of environmental stimuli, rather than the “climate effect” at the class level. Specifically, class-averaged class teacher communication styles can hardly directly alter individuals' already internalized psychological structures, leading to weaker mediating pathways at the class level. This indicates that the impact of class teacher communication style on psychological *suzhi* is more dependent on the individual-individual interaction mode (e.g., communion support in one-on-one communication) rather than the individual-group collective interaction.

Although psychological *suzhi* is a localized construct in China, its core connotation and measurement embody both cultural anchors and cross-cultural relevance. The PSMQ (Hu et al., 2017) used in this study includes items such as “I am very interested in new knowledge” (cognitive trait), “I usually complete my tasks independently” (personality trait), and “I can integrate into and adapt well to my current class environment” (adaptability). These items not only align with the developmental needs of adolescents in the Chinese collectivist context but also cover dimensions shared with Western constructs like psychological capital and psychological resilience.

Discussing this construct in an international journal can offer a “Chinese approach” to global positive psychology research, addressing the limitation of Western positive psychological constructs that primarily focus on state-oriented resources and providing a non-Western perspective on how stable traits mediate the impact of environmental factors. At the same time, it is important to note that the test of psychological *suzhi*'s mediating effect in this study was based on Chinese participants and relied on the role of class teachers as life mentors and the collective management context. The generalization of this conclusion requires caution and further verification in individualistic cultural contexts.

### 4.4 The serial mediating role of basic psychological needs and psychological *Suzhi*

The study's results largely support Hypothesis H4 at L1. Specifically, all L1 serial mediation pathways of BPNs (class teacher communication style → BPNs → psychological *suzhi* → school adjustment) were significant, except the agency → competence → psychological *suzhi* → school adjustment pathway (significant at L2 in M7). Most L1-significant pathways were also L2-significant, with discrepancies primarily in the combined model.

This pathway validates the complementary nature of SDT and psychological *suzhi*-related theories: SDT provides core nourishment for the development of psychological *suzhi* and serves as the process prerequisite for external experiences to be internalized into stable psychological *suzhi*. As (Wang and Zhang 2012a) noted, external influences on individuals' psychological *suzhi* development operate through two pathways: active selection and passive acceptance. The satisfaction of BPNs may act as a navigator in this process. For instance, when a teacher's communication style fosters students' BPNs satisfaction, students are more likely to internalize this adaptive pattern through observational learning and other mechanisms, thereby promoting the enhancement of their psychological *suzhi*.

Consistent with indirect empirical evidence from related constructs (Verleysen et al., 2015; Carmona-Halty et al., 2019), which shows BPN satisfaction correlates with positive psychological resources like psychological capital and resilience, our study extends these insights to psychological *suzhi*. It addresses the scarcity of direct research linking BPNs to psychological *suzhi* (Liu et al., 2024) and further supports the view that BPNs satisfaction may serve as a fundamental motivational basis for the development of positive psychological qualities (Ryan and Deci, 2017).

Each dimension of BPNs plays a distinct role in this correlational serial pathway, their differential “nourishing effects” on psychological *suzhi*: Autonomy satisfaction is associated with a shift from adolescents' passive acceptance of external experiences to proactive engagement (Zhang et al., 2013), which may foster the development of independence (a core personality trait of psychological *suzhi*) and adaptive flexibility (a key component of psychological *suzhi*'s adaptability dimension). Competence satisfaction provides feedback confirming experience effectiveness (e.g., a sense of mastery after completing tasks), which correlates with enhanced cognitive processing fluency and problem-solving flexibility (the core of psychological *suzhi*'s cognitive dimension) and reduced anxiety during adaptation. Relatedness satisfaction creates a safe psychological background for development (Ryan and Deci, 2017), which supports improvements in interpersonal coordination (within psychological *suzhi*'s adaptability dimension) and provides positive emotional feedback for personality development—facilitating the internalization of stable social adaptation patterns.

Notably, the plausibility of this serial pathway is reinforced by the complementary attributes of BPNs and psychological *suzhi*. BPN satisfaction is a dynamic situational variable (e.g., temporary positive feedback from teachers may boost short-term competence satisfaction), while psychological *suzhi* is a stable implicit trait formed by the long-term internalization of repeated need satisfaction (Zhang et al., 2017). This “dynamic process → stable quality” sequence helps explain why class teacher communication style—itself a stable interpersonal tendency (Jiang, 2002)—does not directly correlate with long-term school adjustment, but instead may act through sustained BPNs satisfaction to shape psychological *suzhi*, which in turn shows a more robust correlational link with adaptive outcomes.

From an applied perspective, the serial pathway still highlights practical implications for educational practice. Improving class teachers' communication style (both the communion and agency dimensions) remains a feasible intervention direction: given that communication style's stability provides consistent stimulus for BPN satisfaction (Jiang, 2002), fostering a need-supportive classroom environment through sustained warmth and empathy (communion) and clear, structured guidance (agency) may nourish the cognitive, personality, and adaptability dimensions of psychological *suzhi*—ultimately supporting adolescents' long-term school adjustment.

### 4.5 The moderating role of psychological *Suzhi*

The findings partially supported Hypothesis H5, with clear model-specific patterns: Psychological *suzhi* stably moderated the mediating effect of competence need across both individual and combined models; its moderating effect on autonomy need was significant only in the individual model but non-significant in the combined model; and no significant moderating effect on relatedness need was observed in either model. Psychological *suzhi* is a well-documented moderator in adolescent psychological development (Liu et al., 2024; Chen et al., 2023)—prior research has further confirmed its “stress buffering” role (Pan and Zhang, 2022)—and the present study extends this literature by showing that psychological *suzhi* can moderate specific BPN mediating pathways in the basic psychological need model (Vansteenkiste et al., 2020), though such moderation is not universal across all BPN dimensions.

Notably, the moderating effect of psychological *suzhi* on autonomy need was unstable: it held in the individual model but dissipated in the combined model, suggesting interference from other BPNs. This likely stems from its high covariance with relatedness and competence: once other needs are controlled for, autonomy lacks sufficient unique variance to maintain the moderating effect.

By contrast, its effect on competence need was robust across both models. Simple slope analysis clarified this pattern: low psychological *suzhi* strengthened competence's positive influence on school adjustment, while high psychological *suzhi* weakened and even nullified this effect. This aligns with the notion that stronger psychological *suzhi* provides individuals with sufficient individuality and cognitive qualities to sustain adaptive functioning amid limited external resources (Wang and Zhang, 2012a), whereas lower psychological *suzhi* increases reliance on external competence support. These results highlight the “resource substitution effect” of internal psychological assets, aligning with positive psychology's emphasis on internal strengths in youth development (Xu et al., 2022).

Interestingly, the need-specificity of psychological *suzhi*'s moderating role highlights potential functional differences among the three BPNs in promoting school adjustment. According to prior theoretical discussions (Vansteenkiste et al., 2020), incremental effects and uniqueness are key criteria for a need to be considered basic. Autonomy and competence are closely associated with internal motivation, goal persistence, and self-efficacy—core components of psychological *suzhi*. Therefore, when satisfaction of these needs is low, psychological *suzhi* can play a compensatory role through internal regulatory mechanisms (Li et al., 2012). In contrast, relatedness is more dependent on external interpersonal support, which limits the moderating role of psychological *suzhi* in this pathway. These results empirically support the decision to model the three BPNs separately and further reveal distinct mechanisms through which each need influences school adjustment.

It is important to note the magnitude of the moderating effects: the absolute values of the significant interaction term coefficients were small (ranging from 0.060 to 0.061). Per conventional effect size criteria (Cohen, 2013), such small coefficients suggest that the practical impact of psychological *suzhi* on the mediating pathways of autonomy and competence is limited. However, (Ye and Wen 2013) noted that in psychological research, small effect sizes for moderating effects are relatively common, yet such studies still retain academic value. Furthermore, simple slope analysis revealed that as psychological *suzhi* levels change, the statistical significance of competence's predictive effect on school adjustment undergoes a substantive shift. Thus, it can be argued that the magnitude of this effect size in the present study carries certain theoretical relevance. That said, caution remains warranted to avoid overestimating its substantive significance in real-world educational contexts.

## 5 Limitations and future directions

The primary limitation of this study lies in the method of data reporting. All core variables relied solely on student self-reports. Previous research has shown significant differences between student-reported and teacher-reported data (Brekelmans et al., 2011); even though Harman's single-factor test ruled out severe common method bias, response tendencies (e.g., social desirability) or consistent perceptual biases may still inflate correlations between variables. Thus, incorporating multi-informant data (e.g., teacher-reported communication style) for cross-validation would further enhance the credibility of the conclusions.

A second limitation is the cross-sectional design of the study, which not only prevents the exploration of dynamic reciprocal relationships between BPNs, psychological *suzhi*, and adolescents' school adjustment but also cannot confirm directional causality. For instance, adolescents with better school adjustment may more positively perceive their teachers' communication styles, or those with higher psychological *suzhi* may be more sensitive to need-supportive interactions—such reciprocal influences remain unaddressed in the current design.

A third limitation relates to cultural and sample boundaries. Psychological *suzhi* is deeply rooted in China's quality-oriented education context, and the agency dimension of teacher communication style aligns with traditional Chinese cultural perceptions of teacher authority, which may limit the cross-cultural generalizability of the findings. Additionally, while the sample covers urban, rural, and vocational schools, it does not represent all Chinese adolescent subgroups (e.g., ethnic minorities), which may restrict the broader applicability of the results.

Future research can address these limitations by: (1) adopting a multi-informant (student + teacher) and temporal separation design to reduce common method bias; (2) using a longitudinal design (e.g., 1–2 year follow-ups) to clarify causal directions and investigate dynamic reciprocal relationships among variables; and (3) conducting cross-cultural comparative studies or expanding the sample to include more subgroups, to test the generalizability of the findings and enrich the theoretical framework of teacher-student interaction.

## 6 Conclusion

6.1 Both dimensions of class teacher communication style—communion and agency—significantly and positively predicted adolescents' school adjustment at both the student-level (L1) and class-level (L2).
6.2 The study largely supports three indirect pathways linking class teacher communication style to adolescents' school adjustment, namely the single mediation paths involving basic psychological needs and psychological suzhi, and the serial mediation path from basic psychological needs to psychological suzhi. However, differences exist across basic psychological need types and analytical levels (L1 vs. L2).
6.3 Psychological *suzhi* stably moderated the mediating effect of competence in the relationship between class teacher communication style and adolescents' school adjustment at the L1.

## Data Availability

The raw data supporting the conclusions of this article will be made available by the authors, without undue reservation.

## Footnotes

1 *Communion* = 0.38 × DC+0.92 × CD+0.92 × CS+0.38 × SC−0.38 × SO−0.92 × OS−0.92 × OD−0.38 × DO; *Agency* = 0.92 × DC+0.38 × CD−0.38 × CS−0.92 × SC−0.92 × SO−0.38 × OS+0.38 × OD+0.92 × DO.
